# Vehicle Trajectory Repair Under Full Occlusion and Limited Datapoints with Roadside LiDAR

**DOI:** 10.3390/s25041114

**Published:** 2025-02-12

**Authors:** Qiyang Luo, Zhenyu Xu, Yibin Zhang, Morris Igene, Tamer Bataineh, Mohammad Soltanirad, Keshav Jimee, Hongchao Liu

**Affiliations:** 1 Department of Civil, Environmental and Construction Engineering, Texas Tech University, Lubbock, TX 79409, USA; qiyang.luo@ttu.edu (Q.L.); zhenxu@ttu.edu (Z.X.); morris.igene@ttu.edu (M.I.); tamer.bataineh@ttu.edu (T.B.); mohammad.soltanirad@ttu.edu (M.S.); kjimee@ttu.edu (K.J.); 2 Department of Construction and Transportation Engineering, Ningbo University of Technology, Ningbo 315211, China; yibinzhang@nbut.edu.cn

**Keywords:** roadside LiDAR, full occlusion, limited datapoints, trajectory repair

## Abstract

Object occlusion is a common challenge in roadside LiDAR-based vehicle tracking. This issue can cause variances in vehicle location and speed calculations. This paper proposes a vehicle tracking post-processing method designed to handle full occlusion and limited datapoint conditions. The first part of the method focuses on linking the disconnected trajectories of the same vehicle caused by full occlusion. The second part refines the vehicle representative point to enhance tracking accuracy. Performance evaluation demonstrates that the proposed method can detect and reconnect the trajectories of the same vehicle, even under prolonged full occlusion. Moreover, the refined vehicle representative point provides more stable speed estimates, even with sparse datapoints. This significantly increases the effective detection range of roadside LiDAR. This approach lays a strong foundation for the application of roadside LiDAR in emission analysis and near-crash studies.

## 1. Introduction

High-resolution vehicle trajectory data play a critical role in advancing various fields such as traffic engineering, autonomous driving, and intelligent transportation systems. These data are instrumental for applications such as crash prediction [1,2], traffic density estimation [3], flow monitoring [4,5], car-following analysis [6,7], driver behavior modeling [8,9,10,11], fuel consumption estimation [12,13], adaptive traffic signal control [14], and the development of advanced driver assistance systems (ADAS) [15]. Moreover, they are essential for improving route navigation [16], traffic demand analysis [17], and overall traffic operations in complex urban environments.

Currently, multiple sensor technologies, including cameras, Light Detection and Ranging (LiDAR), radar, and Bluetooth, can provide vehicle trajectory data [5,18,19]. Among these, LiDAR and cameras are known for delivering high-resolution data, which are particularly valuable for detailed trajectory analysis. However, occlusion remains a significant challenge in extracting accurate trajectories, especially in high-traffic intersections or dense urban areas [20,21,22,23]. Occlusion occurs when one vehicle is blocked by another or obscured by objects in the environment, such as the background environment and dynamic moving vehicles [24]. Occlusion has two types, full occlusion and partial occlusion. If full occlusion occurs, the datapoint of a vehicle could totally disappear. In some cases, the full occlusion could be more than one second [25]. When the partial occlusion occurs, the vehicle datapoint could be chopped into two parts or only one part could be detected. Additionally, when the vehicle is far from the LiDAR, only sparse datapoints are captured. This complicates multi-object tracking tasks and reduces data reliability. This paper introduces a novel approach to extract high-resolution vehicle trajectory data using roadside LiDAR. The proposed method addresses two primary challenges: prolonged full occlusion and vehicle representative point refinement under limited datapoints.

The procedure of pre-tracking for roadside LiDAR consists of three main steps: background filtering, object clustering, and identification [16]. To increase the accuracy of the tracking trajectory, the key is to track the same vehicle accurately and continuously [26,27]. A couple of techniques are widely used, including Graph Neural Network (GNN) [1], Joint Probabilistic Data Association (JPDA) [28], Multiple Hypothesis Tracking (MTH) [29], and Hungarian algorithms [30]. GNN utilizes the Euclidean distance to associate the closest vehicle across different point cloud frames. This approach is straightforward to implement and requires relatively low computational resources [31,32]. For example, Zhao [33] employed GNN to track vehicles and pedestrians, achieving a tracking accuracy of approximately 95%. However, GNN often struggles with false tracking and data loss, especially in high-density traffic conditions. To mitigate these issues, Zhang et al. [34] introduced JPDA for tracking in complex traffic environments, which significantly enhanced tracking performance. Despite its advantages, JPDA demands substantial heavy computational power and is prone to combinatorial explosion. Kim and Park [35] introduced an extended Kalman filter (EKF) that incorporates the distance characteristics of LiDAR and radar sensors. By leveraging sensor fusion, their method achieves highly accurate distance estimations. Wu [32] developed a systematic approach for vehicle tracking using roadside LiDAR sensors. Their procedure, structured into five distinct parts, was validated through field testing, demonstrating its effectiveness. Similarly, Vimal Kumar et al. [36] utilized a low-density solid-state flash LiDAR to collect sparse data. By combining the JPDA algorithm with a Kalman filter, they successfully extracted vehicle trajectories, with the results indicating strong performance in trajectory reconstruction using their method. However, when the full occlusion occurs for a long time, such as more than one second, it is still difficult to ensure tracking the same vehicle. It is inevitable for the GNN to generate a new vehicle ID. For the EKF and JPDA, the EKF is a recursive algorithm for estimating the state of dynamic systems by combining predictions with actual observations. Its performance relies on accurate noise models, and under sparse data conditions, noise can cause significant deviations. When sensors lose target information (e.g., prolonged occlusion), the EKF depends solely on predictions, leading to cumulative errors and reduced accuracy. JPDA tracks multiple objects by associating observations with targets using probabilistic hypotheses. Sparse data and occlusion increase uncertainty, degrading association quality and causing trajectory fragmentation after prolonged occlusion. Thus, the EKF and JPDA are unsuitable for prolonged full occlusions. Therefore, a post-tracking method becomes essential to increase the trajectory accuracy.

Another challenge is how to accurately represent the vehicle’s location for association. Two commonly used methods are representative points and boundary boxes. Changes in distance and orientation lead to variations in the vehicle’s shape, resulting in significant differences in the number of datapoints for the same vehicle across different frames. This inconsistency in datapoints affects the stability of the tracking point and boundary box [36]. Zhang et al. [34] used corner points for vehicle tracking. Their results demonstrated that tracking vehicles using corner points significantly enhances accuracy compared to relying on bounding boxes. However, this method relies on enough datapoints. When there are sparse datapoints, this method may not be effective. Later, Wu [37] and Sun et al. [38] further confirmed that, compared to using the vehicle’s center point, employing corner points helps reduce speed estimation errors, making them a more reliable choice for precise tracking. However, the reason why the vehicle’s center point is not considered sufficient as a tracking point lies in the instability of datapoints across frames, particularly in cases of full occlusion or when only a very limited number of datapoints are available. In such scenarios, accurately determining the vehicle’s tracking point becomes challenging, especially during severe partial occlusion. Developing a method to reliably identify the vehicle’s center point using only a limited number of datapoints could effectively resolve this issue.

This post-tracking method is specifically designed to address prolonged full occlusion and scenarios with extremely limited data. The method utilizes information derived from traffic intersection geometry maps, which enriches each vehicle’s data with additional context, such as lane information, etc. (details are provided later). Using the proposed approach, the system identifies and reconnects different vehicle IDs belonging to the same vehicle generated by the full occlusion. Subsequently, the vehicle representative point is adjusted, even under sparse datapoints, to improve tracking accuracy. Experimental results demonstrate that the proposed method is effective in reconnecting trajectories under prolonged full occlusion. Furthermore, the newly adjusted vehicle representative point results in more stable speed estimates.

## 2. Materials and Methods

**Figure 1:**
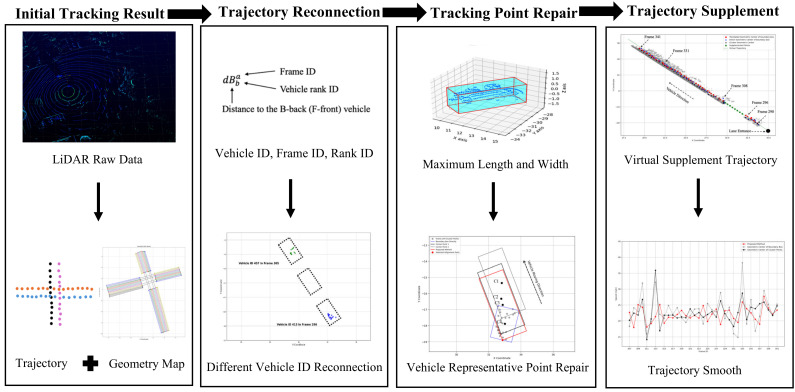
Workflow of the methodology.

The proposed method comprises four main steps: initial tracking, trajectory reconnection, tracking point repair, and trajectory supplementation, as illustrated in Figure 1. In the initial tracking step, various tracking methods exhibit differing levels of performance. However, during prolonged full occlusions, reliably identifying the same vehicle remains a challenge regardless of the tracking method used. Therefore, this study employs the Simple Online Real-time Tracking (SORT) algorithm in the initial tracking phase. The geometric map of traffic intersections, which includes land location in the LiDAR map, is generated by Lin’s method [39]. Based on this geometry map, the coordinate of the lane entrance is acquired, and each vehicle is assigned a lane ID to facilitate subsequent steps. During the trajectory reconnection step, the proposed method links different vehicle IDs that correspond to the same vehicle during periods of full occlusion. The next step involves selecting the most representative frame for the vehicle among the various IDs and using its bounding box to determine the vehicle’s maximum length and width. The representative frame information is then translated along the direction of the vehicle’s predicted trajectory to align with other frames, thereby identifying new representative points for those frames. Finally, a supplement trajectory is generated for the vehicle under prolong full occlusions.

### 2.1. Initial Tracking

The pre-tracking step involves background filtering, object clustering, and object classification. The foundation of this method relies on the initial tracking results and a geometric map. The widely used SORT algorithm was chosen for the initial tracking phase. Since the proposed method utilizes changes in geometric information before and after a vehicle disappears due to full occlusion, a reliable geometric map of the traffic intersection is crucial. This study employs Lin’s method to generate the geometric map [39]. Each vehicle in each frame is assigned a lane ID, such as N_S_1, indicating that the vehicle is moving straight in the No. 1 Northbound Lane.

### 2.2. Trajectory Reconnection

**Figure 2:**
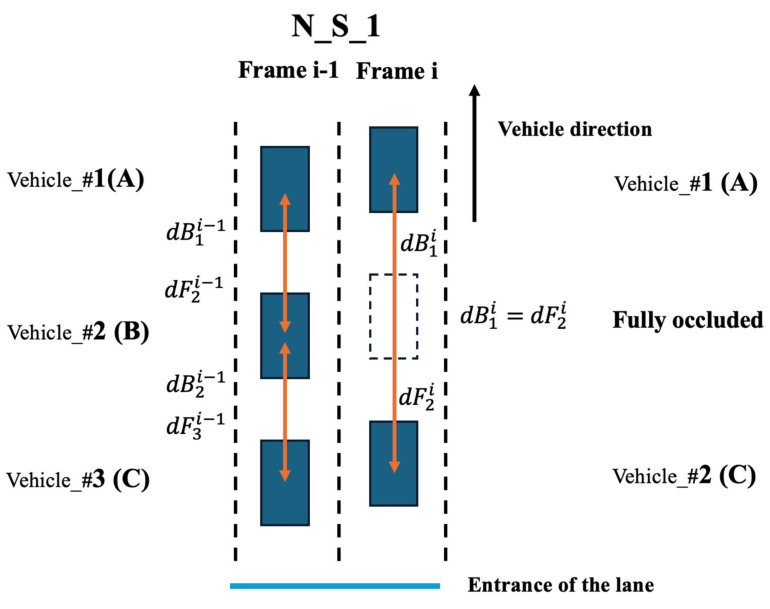
Full occlusion issue.

This step addresses the issue of vehicles under prolong full occlusion. The core principle is to detect changes in distance between each vehicle and its front and rear neighbors in every frame. The process begins by ordering vehicles based on their distance from the lane entrance to assign a rank ID for each frame. For example, as illustrated in Figure 2, in Frame i−1, three vehicles, such as Vehicle #A, Vehicle #B, and Vehicle #C, are present, with Rank ID 1, 2, 3, respectively. For Vehicle #1(A) (Rank ID 1), there is no vehicle in front of it, so it only has the parameter ${dB}_{\beta }^{\alpha }$, which represents the distance to the rear vehicle, as shown in Equation (1). In this step, the center of the cluster is used as the representative point. For Vehicle #2 (B) (Rank ID 2), it has both ${dB}_{\beta }^{\alpha }$ and ${dF}_{\beta }^{\alpha }$, where F stands for “front.” The parameter dF is the distance between the current vehicle and the front vehicle. Similarly, for Vehicle #3 (C) (Rank ID 3), since it is the last vehicle in the lane, it only has dF. In the next frame, due to dynamic factors such as vehicle movement or occlusion caused by vehicles in adjacent lanes, full occlusion occurs. Vehicle B “disappears”, and the rank IDs of the surrounding vehicles (Vehicle A and Vehicle C) and their associated dB and dF values will change accordingly. Specifically, the dB of Vehicle #1 (A) will increase significantly because Vehicle B is no longer present, and now dB measures the distance between Vehicle A and Vehicle C in Frame i. Similarly, the dF of Vehicle A in Frame i will also increase (previously dF measured the distance to Vehicle B  in Frame i−1), as it now measures the distance between Vehicle A and Vehicle C.

To account for dynamic changes such as vehicle speeds and errors in the representative points, a speed and error parameter (γ) is introduced. This parameter is used to evaluate whether the dB value of a vehicle in the current frame falls within an allowable range derived from the dB of the previous frame ($\;[{dB}_{min},\;{dB}_{max}]$). If the dB and dF  exceeds this range (Equations (2) and (3)), full occlusion is determined to have occurred. For instance, in Frame i, the full occlusion of Vehicle B leads to a sharp increase in both ${dB}_{1}^{i}\;$ and ${dF}_{2}^{i}$, surpassing the acceptable range of fluctuations caused by the speed changes of nearby vehicles.

(1) $${dB}_{\beta }^{\alpha }\;and\;{dF}_{\beta }^{\alpha }$$

where dB is the distance between the current vehicle to the following vehicle. dF is the distance between the current vehicle to the following vehicle. α and β are the frame ID and rank ID, respectively.

(2) $${[\;dB}_{i}^{i-1}-\max\left(v_{1}^{i-1},v_{2}^{i-1}\right)*t\;]<{dB}_{i\;}^{i}\;\leq \;[\;{dB}_{i}^{i-1}+\max\left(v_{1}^{i-1},v_{2}^{i-1}\right)*t+\gamma \;]$$

(3) $${[\;dB}_{1}^{i-1}+\max\left(v_{1}^{i-1},v_{2}^{i-1}\right)*t+\gamma \;]<{dB}_{1}^{i}$$

(4) $$\;{\;[\;dF}_{2}^{i-1}+\max\left(v_{1}^{i-1},v_{2}^{i-1}\right)*t+\gamma \;]<{dF}_{2}^{i}$$

where the $v_{\beta }^{\alpha }$ represents the vehicle velocity of Rank ID β in Frame ID α. The t is the time interval 0.1 s. The parameter γ is used to adjust for the possible variation in the vehicle’s center position, and it is set as 0.5 m. The parameter γ is introduced to address the uncertainties associated with estimating the vehicle center and speed variations. This is necessary because, at this stage, the vehicle center is determined based on the cluster center, which may not perfectly represent the actual vehicle center. The true vehicle center should ideally be obtained by incorporating as many vehicles point clouds as possible. If γ is set too small, the system becomes overly sensitive to minor variations in the distance between vehicles, which can result in a higher false positive rate by mistakenly detecting occlusions. Conversely, if γ is too large, the false positive rate decreases, but the false negative rate may increase, meaning the system could fail to detect actual cases of full occlusion. To strike a balance between these risks, γ is set to 0.5. This value effectively manages the trade-off, ensuring that the system maintains sensitivity to true occlusion events while minimizing false detections.

### 2.3. Tracking Point Repair

**Figure 3:**
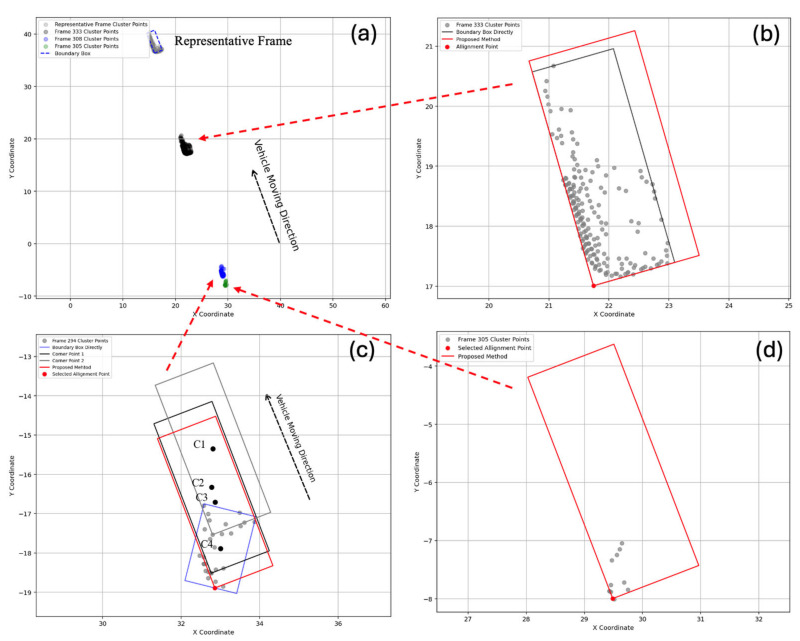
(**a**) The discontinuous trajectory reconnection. (**b**–**d**) Partial occlusion of different datapoints.

This step is designed to address sparse datapoints caused by either partial occlusion or vehicles being too far from the LiDAR sensor. For partial occlusion, there are two scenarios: the first involves a single vehicle being split into two clusters, for which Song’s method is applicable [25]. The second scenario, which is the focus of this paper, occurs when the clustering result contains only a small number of datapoints. After the trajectory reconnection step, datapoints for the same vehicle with different vehicle IDs are consolidated, significantly enhancing the likelihood of obtaining the most comprehensive information about a single vehicle. In previous studies, researchers often relied on corner points, as they are easier to extract than center points. However, if Song’s method [25], shown in Equation (5), is applied, a single clustering result may yield multiple corner points, as shown in Figure 3c. This can lead to errors in determining the corner point of the vehicle, thereby affecting speed calculations. Similarly, if Zhang’s method [34] is used to locate the center point, it also requires enough datapoints. Furthermore, neither Song’s nor Zhang’s methods are suitable for situations with very sparse datapoints, as illustrated in Figure 3d.

(5) $$\beta =\max\left({tan}^{-1}\left(\frac{Y_{m}-Y_{n}}{X_{m}-X_{n}}\right)\right)-\min\left({tan}^{-1}\left(\frac{Y_{m}-Y_{n}}{X_{m}-X_{n}}\right)\right),\;n\epsilon \;NV\;\&\;n\neq m\;$$

where slope is denoted as α. The angle β between max α and min α can then be calculated. Here, β is set as $5^{{}^{\circ}}$. If the $90^{{}^{\circ}}-\gamma \leq \;\beta \;\leq \;90^{{}^{\circ}}+\gamma$, point m is selected as the corner point.

To address this limitation, a novel approach is proposed, which translates the boundary box of the representative frame to encapsulate the remaining frames and refine the vehicle’s center point. The bounding box is selected from the representative frame with the most comprehensive data collected for a specific Vehicle ID during the trajectory reconnection step. Figure 3a shows a known representative frame, which is then used to process three different cases of data density: high, medium, and low, corresponding to Figure 3b, Figure 3c, and Figure 3d, respectively. To identify alignment points, different methods are applied based on the density of datapoints. The pseudo-code is illustrated in Algorithm 1. For cases with a moderate number of datapoints ($\frac{N_{j}}{N_{k}}>0.6$), the point within the boundary box that is closest to the LiDAR and the lane entrance is selected as the alignment point for the representative frame. For cases with a high number of datapoints ($\frac{N_{j}}{N_{k}}\leq 0.6$), the alignment point is chosen from the cluster data, specifically the point closest to the LiDAR and the lane entrance.
**Algorithm 1.** Selection of the aligned point.$\mathrm{Input}\;j,\;k,\;N_{j},\;N_{k},\;(x_{0},y_{0})$$,\;(x_{e},y_{e})$$,\;w_{1}$, $w_{2}$$\mathrm{Note}:\;j\;\mathrm{is}\;\mathrm{the}\;\mathrm{target}\;\mathrm{frame}\;\mathrm{ID},\;k\;\mathrm{is}\;\mathrm{the}\;\mathrm{representative}\;\mathrm{frame}\;\mathrm{ID},\;N_{j}\;\mathrm{is}\;\mathrm{the}\;\mathrm{datapoint}\;\mathrm{number}\;\mathrm{of}\;\mathrm{frame}\;j,\;N_{k}\;\mathrm{is}\;\mathrm{the}\;\mathrm{datapoint}\;\mathrm{number}\;\mathrm{of}\;\mathrm{frame}\;k.\;\left(x_{e},y_{e}\right)$$\;\mathrm{is}\;\mathrm{the}\;\mathrm{coordinate}\;\mathrm{of}\;\mathrm{the}\;\mathrm{entrance}\;\mathrm{of}\;\mathrm{the}\;\mathrm{lane},\;\left(x_{0},y_{0}\right)$$\;\mathrm{is}\;\mathrm{the}\;\mathrm{coordinate}\;\mathrm{of}\;\mathrm{the}\;\mathrm{LiDAR},\;\mathrm{weighted}\;\mathrm{parameter}\;w_{1}=w_{2}=0.5$.BeginIf $\frac{N_{j}}{N_{k}}\leq 0.6$, the selected aligned point is the cluster point that is closest to both the LiDAR and the lane entrance. P is the set of points in the cluster points. 
($x^{*},y^{*}$) = $\underset{(x_{i},y_{i})\epsilon P}{argmin}$ [$w_{1}\cdot \sqrt{{\left(x_{i}-x_{0}\right)}^{2}+{\left(x_{i}-y_{o}\right)}^{2}}+w_{2}\cdot \sqrt{{\left(x_{i}-x_{e}\right)}^{2}+{\left(x_{i}-y_{e}\right)}^{2}}$]2.If $\frac{N_{j}}{N_{k}}>0.6$, the selected aligned point is the corner point of the boundary box that is closest to both the LiDAR and the lane entrance. B is the set of corner points in the boundary box. 
($x^{*},y^{*}$) = $\underset{(x_{i},y_{i})\epsilon B}{argmin}$ [$w_{1}\cdot \sqrt{{\left(x_{i}-x_{0}\right)}^{2}+{\left(x_{i}-y_{o}\right)}^{2}}+w_{2}\cdot \sqrt{{\left(x_{i}-x_{e}\right)}^{2}+{\left(x_{i}-y_{e}\right)}^{2}}$]3.End ifEND

### 2.4. Virtual Trajectory Supplement

This step aims to supplement the “missing” trajectory caused by full occlusion. Following the trajectory reconnection and trajectory point relocation steps, the frame ID and positions of the vehicle before and after the full occlusion have been identified. This step involves connecting the trajectory points associated with the two previously identified vehicle IDs. Using the geometric map, a reference trajectory is provided. The positions of the two frame IDs are then projected onto this virtual trajectory. Interpolation is applied to reconstruct the “missing” portion of the trajectory. The process is illustrated in Algorithm 2. The first step is to project the coordinates of the two vehicle IDs ($x_{a}^{m},\;y_{a}^{m}$) and ($x_{b}^{n},\;y_{b}^{n}$), both before and after disappearance, onto the predicted trajectory to obtain (${x\prime }_{a}^{m},\;{y\prime }_{a}^{m}$) and (${x\prime }_{b}^{n},\;{y\prime }_{b}^{n}$). Then, apply linear interpolation to compute the “missing” segments of the trajectory.
**Algorithm 2.** Generating the virtual trajectory.Input: a, b, m, nNote: a and b are two vehicle IDs belonging to the same vehicle in frame m and frame n, and m<n.BeginFind the coordinate ($x_{a}^{m},\;y_{a}^{m}$) and ($x_{b}^{n},\;y_{b}^{n}$)2.Project the ($x_{a}^{m},\;y_{a}^{m}$) and ($x_{b}^{n},\;y_{b}^{n}$) to the reference lane provided by the geometry map, and acquire the (${x\prime }_{a}^{m},\;{y\prime }_{a}^{m}$) and (${x\prime }_{b}^{n},\;{y\prime }_{b}^{n}$)3.Calculate t=(n−m)·0.1, for each intermediate frame i,  where m<i<n, calculate the position $\left(x_{,\;}^{i}y^{i}\right)$ using linear interpolation along the virtual road segment: ${{x^{i}=x}^{\prime }}_{a}^{m}+\frac{i-m}{n-m}\cdot \left({{{x^{\prime }}_{b}^{n}-x}^{\prime }}_{a}^{m}\right),{{y^{i}=y}^{\prime }}_{a}^{m}+\frac{i-m}{n-m}\cdot \left({{{y^{\prime }}_{b}^{n}-y}^{\prime }}_{a}^{m}\right)$END

### 2.5. Data Collection

**Figure 4:**
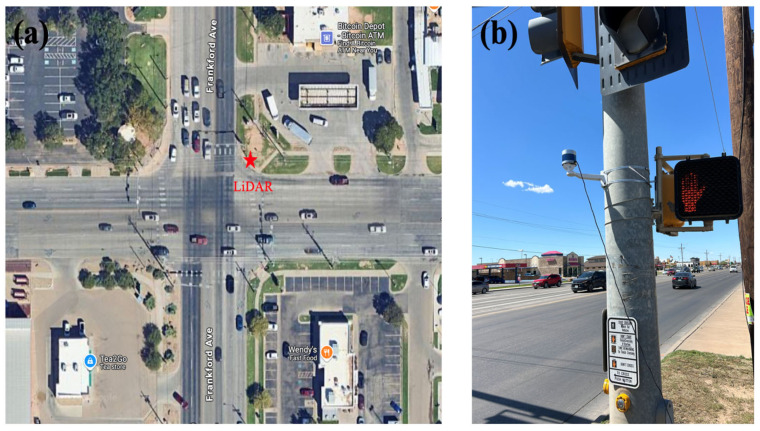
(**a**) Area of study. (**b**) A VLP-32 LiDAR sensor mounted on a traffic signal pole.

In this study, the VLP-32 LiDAR sensor, is sourced from Velodyne Lidar, Inc., San Jose, CA, USA, was used for data collection. The sensor has 32 channels with vertical scan angles from −15° to +15° and a 360° horizontal scan range. Operating at a rotational frequency of 10 Hz, it captures 10 frames per second, with each frame containing approximately 600,000 3D points. An urban high-traffic intersection, Frankford Ave and 4th St, in Lubbock, Texas, in the USA was selected for the performance evaluation of the proposed method, as shown in Figure 4. The data were collected in the peak hours from 4 p.m. to 6 p.m.

## 3. Results

### 3.1. Full Occlusion Detection

**Figure 5:**
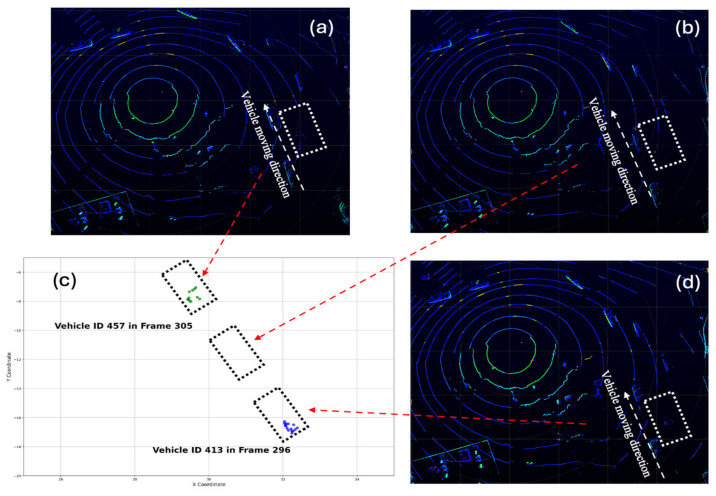
A vehicle trajectory connection (**a**) after the full occlusion (**b**) during the full occlusion (**c**) comparison before and after the full occlusion (**d**) before the full occlusion.

Figure 5 illustrates an example of the results for full occlusion detection. In this case, the vehicle is fully occluded between frame ID 296 and frame 305, disappearing for a total of 9 frames (0.9 s). This duration is sufficient for SORT to generate a new vehicle ID. However, using the proposed method, vehicle ID 457 and vehicle ID 413 are identified as belonging to the same vehicle. The underlying principle is that when vehicle ID 457 disappears in frame 297, the dB of the preceding vehicle and the dF of the following vehicle increase significantly. These increases exceed the normal ranges of dB and dF, thereby triggering full occlusion detection. This example demonstrates the effectiveness of the proposed method in detecting prolonged full occlusion.

### 3.2. Tracking Point Refinement and Virtual Trajectory Generation

**Figure 6:**
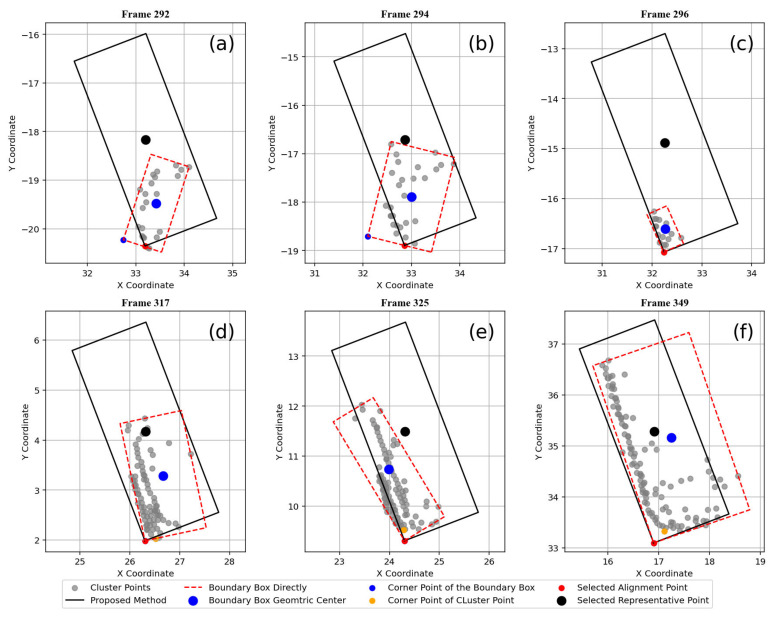
Vehicle representative repair for (**a**–**f**) frames 292, 294, 296, 317, 325, and 349.

Figure 6 illustrates the results of tracking point refinement, where the proposed method is applied to identify representative points under a varying number of datapoints. As shown in Figure 6a–c, directly using the boundary box to represent the tracking point is unavailable when the number of datapoints is small, let alone relying on the cluster center. This is because, in cases of partial occlusion or when the vehicle is far from the LiDAR, the number of datapoints per vehicle is significantly reduced. By employing the proposed method (NjNk>0.6), a relatively accurate vehicle center can be determined, especially when the data volume is extremely low, as demonstrated in Figure 6c. For situations where the number of datapoints is substantial, the proposed method can still provide a relatively accurate vehicle position. This is because partial occlusion persists in these cases; however, it is less severe. Moreover, by translating the boundary box of the representative frame, a more accurate vehicle center can be acquired.

**Figure 7:**
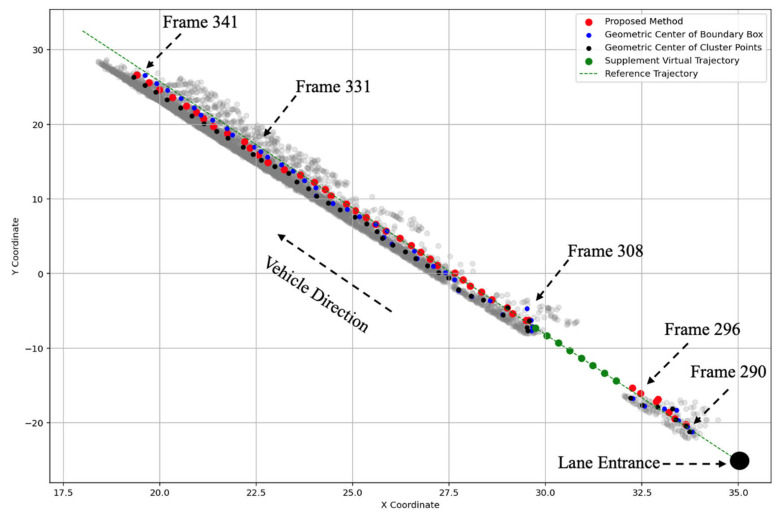
Different representative point location adjustments.

**Figure 8:**
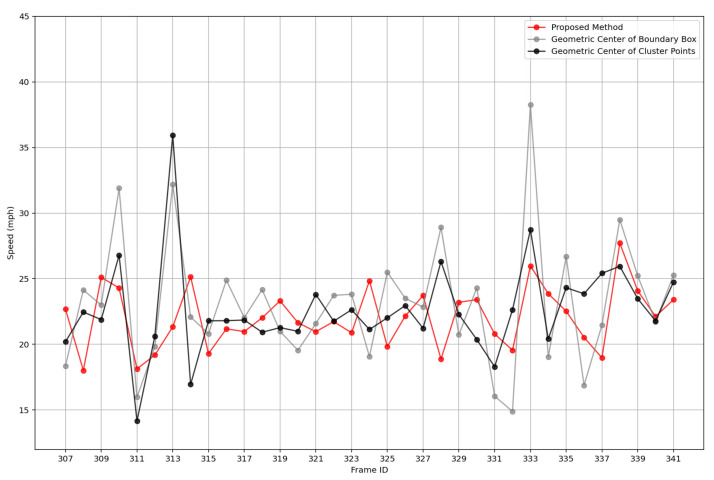
Velocity comparison with different representative point methods.

Figure 7 and Figure 8 present a comparison of the trajectories of representative points and their corresponding speeds using the cluster center, boundary box, and the proposed method, from frame 290 to frame 341. For points with a small number of LiDAR detections on a single vehicle, the representative points provided by the proposed method are relatively stable and continuous, with minimal deviation from the reference trajectory (e.g., frame 296 and frame 308). For points with a larger number of LiDAR detections on a single vehicle, the speeds generated by the proposed method are more stable compared to the other two methods, exhibiting lower speed variance. Moreover, the proposed method ensures that the speed is less influenced by fluctuations in the data volume.

### 3.3. Compared to the State-of-the-Art Method

**Figure 9:**
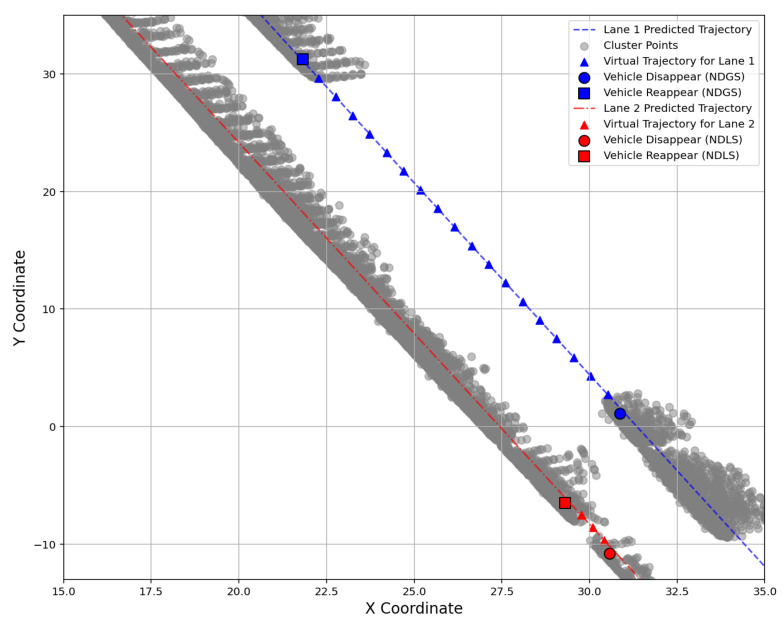
Multiple vehicle trajectory Reappear for prolonged full occlusion.

To validate the proposed method, we selected the traffic flow in four directions of a traditional intersection as a test scenario, consisting of straight roads and curved roads (left and right turns). To evaluate the method’s performance in detecting prolonged full occlusion, we divided the discontinuous trajectories into two categories: those lasting less than 0.5 s (NDLS) and those greater than 1.0 s (NDGS), as shown Figure 9. The results were compared with the state-of-the-art methods proposed by Zhao [33] and Song [25], as shown in Table 1 and Table 2. To the best of our knowledge, Zhao’s [33] and Song’s [25] methods represent some of the most advanced approaches currently available.

The proposed method outperforms Zhao’s method [33] in reconstructing long-term full occlusions on straight roads, particularly in cases of prolonged full occlusions, as shown in Table 1 and Table 2. When compared to Song’s method [25], the performance on straight roads is similar for discontinuous trajectories shorter than 0.5 s. However, for discontinuous trajectories lasting longer than 1.0s on straight roads, the proposed method demonstrates superior performance. This improvement can be attributed to two key reasons: 1. Song [25] detects full occlusion within a spatial range rather than on a lane-by-lane basis. 2. Song’s [25] time threshold for detecting full occlusion is limited to 1.5 s, after which the search is terminated. Since Song’s [25] approach relies on a range-based detection rather than a lane-specific one, its performance on curved roads is inferior to the proposed method. This is expected, as range-based searches are more susceptible to interference from adjacent lanes. In contrast, the proposed method performs lane-by-lane detection, effectively mitigating this issue. However, as this method is based on a geometric map, it may encounter challenges during turns, including traditional left and right turns, as well as special cases such as S-curves and other irregular road geometries. The accuracy of the predicted trajectory plays a significant role in the algorithm’s performance under these conditions. When a vehicle turns, there are different potential turning scenarios. If a prolonged full occlusion occurs during a turn, the algorithm may face risks of failure. This is because the method assigns a lane ID to the vehicle in each frame, and inaccurate trajectory prediction will affect the judgment of the vehicle’s lane. This explains why the proposed method consistently performs better on straight roads than on curved roads: the predicted trajectory for straight roads is easier to obtain and more reliable compared to that for curved roads.

## 4. Discussion

**Figure 10:**
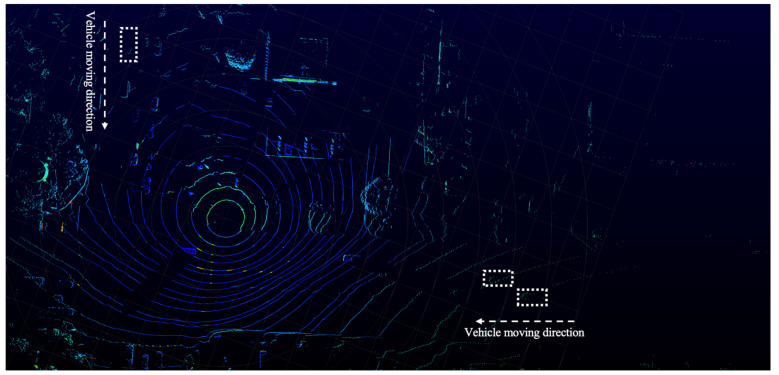
LiDAR effective detection range increase.

The proposed method proves to be highly effective in addressing the challenge of full occlusion at busy intersections. It successfully connects different vehicle IDs belonging to the same vehicle, even during prolonged full occlusion. In terms of finding representative points, the method is capable of accurately tracking the center of the vehicle, even when datapoints are extremely limited. This capability not only enhances tracking accuracy and stability but also significantly extends the effective detection range of LiDAR, as illustrated in Figure 10. Traditional methods often fail to track vehicles under such sparse data conditions, but the proposed method demonstrates remarkable success.

However, certain factors may influence the accuracy of the proposed method. The first is the geometric map. While the examples in this study are based on straight roads, the method can be applied to curved road sections as well. The challenge, however, lies in accurately defining road boundaries, as the method depends on the geometric map to provide lane information for vehicles. The second limitation is related to lane changes. The proposed method cannot detect lane change behaviors. Nevertheless, since the method is designed for intersections with heavy traffic, lane change occurrences are relatively rare in this scenario due to the small gaps between vehicles and the limited space available for lane changes.

Although the proposed method utilizes the VLP-32 LiDAR, it is applicable to different ranges of LiDAR devices, including both VLP-64 and VLP-16. This is because the principle behind detecting full occlusion in the proposed method is based on the impact of the occluded vehicle on the preceding and following vehicles during prolonged full occlusion, which is independent of the LiDAR’s frequency. Moreover, regardless of the LiDAR’s frequency, vehicles are prone to experiencing partial occlusion and full occlusion during movement. In such scenarios, the proposed method’s trajectory reconnection and tracking point repair functions effectively, making it suitable for LiDAR devices across various frequency ranges.

With the advancement of Artificial Intelligence (AI), it has become a powerful tool for enhancing roadside LiDAR-based vehicle tracking. Future work can leverage AI to improve performance in real-time prediction of missing trajectories, refined trajectory interpolation, and adaptive occlusion recovery beyond predefined geometric maps: 1. Real-Time Prediction of Missing Trajectories: Generative AI models can predict missing trajectory segments by learning from historical and real-time roadside LiDAR data, complementing trajectory reconnection and reducing errors from prolonged occlusions. 2. Refining Trajectory Interpolation: By using probabilistic diffusion methods, foundation models can refine trajectory interpolation while addressing noise and sparse data, improving tracking accuracy and speed estimates. 3. Adaptive Occlusion Recovery: Generative models can dynamically adapt to various road geometries and environmental conditions without relying solely on predefined maps, effectively handling occlusions in challenging layouts like sharp turns or complex intersections. Integrating AI into roadside LiDAR-based tracking offers a promising direction for future research and enhances the robustness of the proposed method under sparse data and prolonged occlusion scenarios.

## 5. Conclusions

This paper introduces a novel vehicle trajectory supplementation method designed specifically for high-density intersections, addressing the situation of tracking vehicles in prolong full occlusion and limited datapoint situations. This method improves the relia-bility of vehicle tracking in high-density intersections. The main contributions of this work are summarized as follows: 1. Improved trajectory accuracy under full occlusion: This method can track vehicles even in cases where full occlusion causes large track gaps. 2. Vehicle center localization under sparse data conditions: The center of the vehicle can be accurately identified even when the LiDAR data points are limited due to partial occlusion. 3. Improved practicality of LiDAR at long distances: This method extends the effective detection range of LiDAR and enables vehicle tracking even at the outer limit of the LiDAR range (where the single vehicle data points are significantly reduced). This work offers potential benefits for a variety of transportation applications, including traffic counting, vehicle speed tracking, adaptive traffic signal control, and traffic safety analysis. However, certain limitations of the proposed method must be acknowledged. There are specific scenarios that the method cannot effectively handle, and developing solutions for these situations will be an avenue for future research. Previous studies have shown that the use of multiple sensors can improve object tracking performance. While this study focused only on vehicle detection and tracking using roadside LiDAR sensors, integrating data from other sensors such as cameras and radars may improve accuracy in specific situations. Exploring the fusion of data from different traffic sensors to further improve tracking accuracy provides an exciting direction for future research.

## Figures and Tables

**Table 1:** Data processing results with different methods for short discontinuous trajectories.

Type	NDLS	Percentage of Fixed Occlusion with Zhao [33]	Percentage of Fixed Occlusion with Song [25]	Percentage of Fixed Occlusion with the Proposed Method
Straight Road	92	43.48%	83.70%	94.57%
Curved Road	56	None	62.50%	82.14%

NDLS: number of detected discontinuous trajectories less than 0.5 s.

**Table 2:** Data processing results with different methods for long discontinuous trajectories.

Type	NDGS	Percentage of Fixed Occlusion with Zhao [33]	Percentage of Fixed Occlusion with Song [25]	Percentage of Fixed Occlusion with the Proposed Method
Straight Road	45	22.22%	64.44%	91.11%
Curved Road	33	None	30.30%	81.82%

NDGS: number of detected discontinuous trajectories greater than 1.0 s.

## Data Availability

Data will be made available on request.
